# Interrupting behaviour: Minimizing decision costs via temporal commitment and low-level interrupts

**DOI:** 10.1371/journal.pcbi.1005916

**Published:** 2018-01-16

**Authors:** Kevin Lloyd, Peter Dayan

**Affiliations:** Gatsby Computational Neuroscience Unit, London, United Kingdom; Leiden University, NETHERLANDS

## Abstract

Ideal decision-makers should constantly assess all sources of information about opportunities and threats, and be able to redetermine their choices promptly in the face of change. However, perpetual monitoring and reassessment impose inordinate sensing and computational costs, making them impractical for animals and machines alike. The obvious alternative of committing for extended periods of time to limited sensory strategies associated with particular courses of action can be dangerous and wasteful. Here, we explore the intermediate possibility of making provisional temporal commitments whilst admitting interruption based on limited broader observation. We simulate foraging under threat of predation to elucidate the benefits of such a scheme. We relate our results to diseases of distractibility and roving attention, and consider mechanistic substrates such as noradrenergic neuromodulation.

## Author summary

Animals should ideally be able to monitor all relevant aspects of their environment constantly and be ever prepared to alter their course of action in the face of unexpected change. However, the impractically high costs of continual monitoring and deliberation mean that a more realistic strategy is required. Here, we explore a solution in which an animal makes provisional commitments to a temporally-extended action while maintaining the ability to interrupt this behaviour prematurely on the basis of more limited sensing. We demonstrate the benefits of such a scheme through the example of foraging under predation risk, and propose a simple mechanism for implementing interruption. We suggest possible relationships between these results and neural substrates, particularly norepinephrine, and also highlight potential relevance to diseases of distractibility.

## Introduction

It might seem optimal for decision-makers to be constantly open to all sources of potential information and to be able to change their course of action at a moment’s notice. However, a range of physical and computational constraints makes such a prospect infeasible.

In terms of sensation, relevant information may not always be accessible when the agent engages in an activity that naturally restricts sensory access. In other words, agents create *partially observable* environments for themselves, even when those environments could be more fully observable [1]. Furthermore, even if information is available in principle, limited computational resources mean that only a subset of this information can in practice be subject to enhanced perceptual or central processing. These considerations lead to forms of selective attention [2–4].

Constant reassessment of decisions seems similarly impractical. Working out the correct course of action in terms of maximizing expected utility is computationally demanding, requiring consideration of the possible consequences of each current option. Even when only approximate optimality or satisficing is sought [5, 6], it is desirable to minimize the frequency with which such costly operations take place. Equally, switching between courses of action likely incurs overheads in terms of time or even energetic costs [7–9]. The obvious alternative is to commit to particular courses of action or undertakings for extended periods of time, limiting the frequency of expensive deliberation steps.

There is a synergy between temporal commitment and selective attention: during extended periods of performing a single undertaking, the decision-maker could focus on just that part of the external information which is strictly relevant. The trouble with this strategy is that of being insufficiently *reactive*, potentially leading to failures to respond appropriately to unexpected opportunities or threats [10, 11]. Here, we therefore consider the intermediate strategy of making provisional temporal commitments while also allowing ongoing behaviour to be *interrupted*. Such interruptions are occasioned by a strictly limited monitoring process that collects broader, but lower quality, information about the environment. This combination balances the desire to minimize decision-making costs with the ability to respond in a timely and appropriate way to changing requirements. The possibility of interruption would be a form of strategic hedge against potentially changing circumstances.

Such considerations are venerable: the need to plan and act in a way that is responsive to real-world demands, but which is sensitive to constraints on time and resources, has been extensively discussed in the artificial intelligence and related literatures [1, 12, 13]. This includes recognition of the benefits of planning with temporally-extended units of activity in terms of search complexity [14, 15], and exploration of issues surrounding replanning, such as the need for monitoring to determine whether replanning is required [16], and the use of contingency plans that specify—to varying degrees of detail—what to do under different future scenarios [17, 18]. Similarly, conventional computers use interrupts for a variety of purposes, including allowing external events, such as the press of a key on a keyboard, to prompt the central processing unit to set aside its current activity; this is an alternative to engaging in constant ‘polling’ of the relevant devices (see, e.g., [19]).

In the natural world, these forms of interruption can be expected to apply over the timescale of a behaviour such as a bout of foraging (i.e., over multiple seconds or even minutes). In this paper, we use a detailed example to examine this particular timescale. By contrast, previous work on natural aspects of interruption has focused on day-long [20] and sub-second [21, 22] interruption. In both of these cases, norepinephrine (NE), a neuromodulator involved in arousal, vigilance and attention [23, 24], has been implicated as a medium for an interrupt signal, putatively in virtue of reporting forms of unexpected uncertainty [25, 26].

Illustrating the operation of such a scheme requires an environment with costs and benefits for action, and both uncertainty and change. We consider the case of foraging under predation risk [27, 28], in which a decision-maker faces various potential tradeoffs between energetic gain and danger [29]. This offers a flexible framework to explore choices of existential importance, for instance between a habitat that promises a high rate of gain but a high risk of predation, and one that promises a lower rate of gain but less risk. We also explore the case that foraging is incompatible with high quality, active assessments of threats.

The rest of the paper is organised as follows. We first consider a simple motivating example that illustrates the basic ideas. We then detail the full model, describing the nature and capacities of a foraging agent and the environment in which it operates. We proceed to explore optimal behaviour in the full model with and without the possibility of an interrupt, and with and without decision costs. We also examine the possibility of using a computationally simpler interruption scheme. Finally, we discuss possible links to NE and diseases of interruptibility.

### Simple foraging example

The full model of foraging under predation risk that we consider below includes a number of detailed components. Therefore, in order to illustrate and provide intuition for the workings of interruption, we start with a simple, stripped-down model.

**Fig 1:**
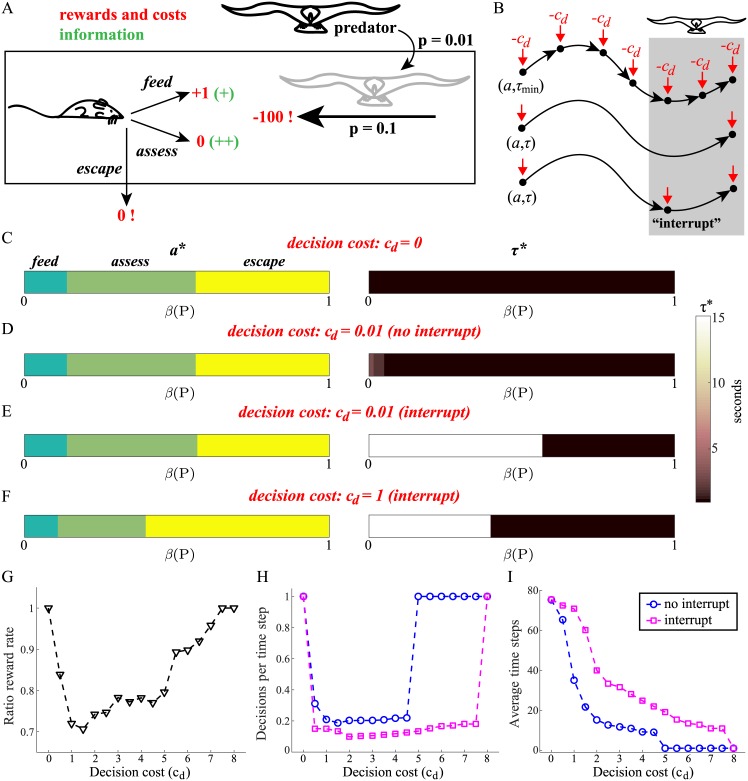
The ability to interrupt behaviour promotes temporal commitment. (A) Simple foraging example. A rat chooses to *feed* (reward +1 per time step), *assess* (reward 0), or *escape* (reward 0), in a habitat where the predator arrives with probability 0.01 per time step and then stays. Although *assess* is not rewarded, it provides more reliable information about whether the predator has arrived (++) than *feed* (+). If the predator has arrived, the probability that the rat gets caught if it remains in the habitat is 0.1 per time step. The decision process terminates (indicated by a **!**) either when the rat is caught (reward −100) or chooses to *escape*. (B) If the animal makes decisions at a high frequency, for example committing to an action *a* for only the minimum amount of time *τ*_min_ (top), it will be able to respond to unexpected changes (such as the arrival of the predator; grey box) but will also incur large decision costs; each decision incurs a cost of *c*_*d*_. Committing for a longer duration *τ* has the advantage of reducing decision costs (middle), but is risky—the predator may arrive before the action terminates—unless the animal has the option to interrupt its behaviour at an earlier time (bottom). (C–F) Optimal policies, comprising an optimal action a* (left) and corresponding optimal duration τ* (right), for four different conditions. (C) No decision cost, *c*_*d*_ = 0. The optimal duration τ* is always as short as possible, meaning that the animal will be best able to respond appropriately if the predator arrives. (D) Decision cost *c*_*d*_ = 0.01, no interrupt. Optimal durations are essentially still as short as possible; the animal pays the greater cost of making high-frequency decisions in order to maintain its responsiveness. (E) Decision cost *c*_*d*_ = 0.01, interrupt. With the capacity to interrupt ongoing behaviour, the animal now selects long durations for *feed* and *assess*; the interrupt allows the animal to minimize its decision costs and also remain reactive, since it can always interrupt and make a new decision when required. (F) When the decision cost is increased further, *c*_*d*_ = 1, the shape of the policy changes so that *escape* is more predominant. (G) Ratio of average total amount of reward per trial (non-interrupt/interrupt) for different decision costs. For each decision cost, optimal non-interruptible and interruptible policies performed 1000 trials of the foraging task. (H) Average number of decisions per time step, and (I) average number of time steps of the optimal non-interrupible (blue circles) and interruptible (magenta squares) policies as a function of decision cost.

Consider an animal (e.g., a rat) foraging in a habitat (Fig 1A) in which a predator (e.g., a hawk) arrives with probability 0.01 per unit time. The animal has to choose between three actions: (i) to continue to *feed*; (ii) to stop feeding to *assess* whether a predator is present; or (iii) to *escape*. Opting to *feed* has the benefit of gaining resources (worth 1 unit of reward per unit time), but has the drawback of providing only poor information (+) about whether the predator is present (e.g., because it restricts the animal’s field of view). Furthermore, if the predator is present, then the animal gets caught with probability 0.1 per unit time, incurring a cost of −100 units of reward, and causing the task to terminate. By contrast, *assess* provides the animal with improved information (++) about the presence of the predator, but has reward 0 per unit time. The exact details of how the quality of information is varied shall be described below, but the important point here is that *feed* and *assess* have distinct informational consequences. Finally, choosing to *escape* leads to a location that is safe, but does not permit foraging, thus gaining 0 reward; it also causes the task to terminate. The animal’s assumed task is to maximize the expected sum of its undiscounted future rewards (we consider the more conventional case of long run discounted rewards when we amplify this simple example).

The animal here has to negotiate two fundamental tradeoffs. One is between the rewards that can be directly obtained by choosing to *feed* and the improved information that can be obtained by choosing to *assess*. The second tradeoff is between future possible rewards that may be obtained by remaining in the habitat, and the sacrifice of those rewards in favour of safety by choosing to *escape*.

The final detail of the example is that each time the animal makes a choice about what to do, it selects both an action *a* (i.e., *feed*, *assess*, or *escape*) and a duration *τ* with which to perform it. Critically, each time the animal makes a decision it may incur a *decision cost*, *c*_*d*_ ≥ 0, which summarizes the various computational demands associated with such a decision; as discussed above, these may include planning and/or switching costs. If this decision cost is non-negligible, the animal additionally faces a tradeoff between reducing decision costs by making a prolonged temporal commitment to an action—i.e., reducing decision frequency by choosing a long duration *τ*—and being able to respond quickly if it is likely that the predator has arrived (Fig 1B).

Whether or not the animal is able to *interrupt* its ongoing behaviour is a critical determinant of its optimal policy. Here, a policy is a mapping between the degree to which the animal believes that the predator has arrived/is present, *β*(*P*), and an action-duration pair (*a*, *τ*). Fig 1C–1F display optimal policies, comprising optimal actions a* (left) and associated optimal durations τ* (right), for four different cases. The first (Fig 1C) is the optimal policy when there is no decision cost, *c*_*d*_ = 0. In terms of actions, the animal chooses to *feed* when it is unlikely that the predator has arrived (*β*(*P*) low), to *escape* when this is more likely than not (*β*(*P*) > 0.5), and to *assess* otherwise. More importantly for our purposes is the observation that optimal durations τ* are uniformly chosen to be as short as possible. Since making a decision has no cost, doing so at the highest possible frequency is the best way to ensure that the animal is best able to respond if it thinks the predator may have arrived.

Next, we consider the case where there is a small decision cost, *c*_*d*_ = 0.01, and the animal is unable to interrupt its own activity (Fig 1D). That is, if the animal chooses to engage in an action for *τ* seconds, it will be fully committed to performing the action for that duration. Here, there is no change in choice of actions, and very little change in duration except a slight increase in τ* for *feed* when the predator is very unlikely to have arrived. The low durations here, even though this entails a high frequency of decisions, mean that the animal is willing to pay these decision costs in order to remain responsive to possible threat.

In Fig 1E, the decision cost remains the same (*c*_*d*_ = 0.01), but the animal is now able to interrupt its behaviour. Interrupts also occur as a function of *β*(*P*), but (a) as noted, the information available to change *β*(*P*) is of lower quality during *feed* than *assess*; and (b) we assume that interruption itself is *free* (although there is then a standard decision cost associated with the necessary re-planning). Again, we see no change in which actions are chosen, but now both *feed* and *assess* are chosen to have the *longest* possible duration (in this example, there is no advantage to the animal of choosing to spend longer on *escape*). Since the animal can always interrupt itself, making such provisional commitments means that it can minimize decision costs by interrupting and making a new decision only when strictly necessary.

Finally, if the decision cost is increased further, changes in the pattern of optimal actions are observed. Fig 1F shows the optimal policy for *c*_*d*_ = 1, showing an increased propensity of the animal to *escape*. The alternatives are increasingly disfavoured by the animal not because of any change in risk of predation—this is unchanged—but because of the increased expense of making on-going decisions if it remains in the habitat.

The preceding discussion suggests that the ability to interrupt behaviour should be advantageous: it allows an animal to minimize decision costs by making provisional commitments to temporally-extended actions, while maintaining its ability to respond to changes in the environment. Indeed, an advantage in terms of rate of rewards is observed (Fig 1G). When there is no decision cost (*c*_*d*_ = 0), the ratio of reward rates (non-interruptible/interruptible) is 1, reflecting the fact that an animal would perform equally well whether or not it is equipped with an interrupt. However, as *c*_*d*_ increases, this ratio decreases, reflecting a reward advantage when the option to interrupt is available. Beyond a critical value, the ratio reverts to 1, with the animal deciding to escape immediately.

This advantage in terms of reward rate arises from the reduction in the frequency of decisions when the animal is able to interrupt (Fig 1H). This reduced decision rate makes it worthwhile for the animal to spend more time (safely) foraging in the environment (Fig 1I), thereby increasing its haul of rewards. The advantage disappears when planning is so expensive that the animal *escape*s immediately. However, the cost at which this occurs is lower when interruption is not possible (cf. Fig 1H and 1I).

## Methods

Our simple example serves to demonstrate the following basic points: when making decisions is costly, the ability to interrupt on-going behaviour promotes provisional commitments to temporally-extended actions, and this ability confers a demonstrable advantage in terms of rewards. However, it makes a number of unrealistic assumptions, notably a single possible interaction with the predator.

**Fig 2:**
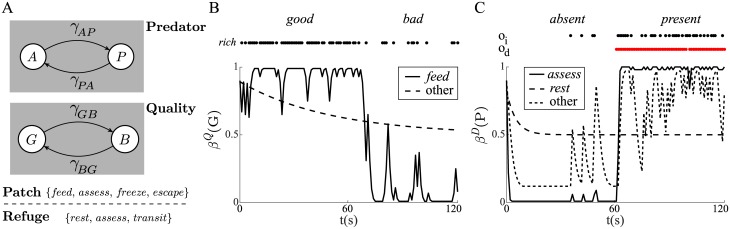
Model of foraging under predation risk. (A) The animal can either remain safely in its refuge or forage outside in a ‘patch’ location. The payoff for foraging depends on the habitat quality, which varies between *good* (*G*) and *bad* (*B*) states with transition rates (*γ*_*GB*_, *γ*_*BG*_), and whether a predator is *absent* (*A*) or *present* (*P*), as determined by transition rates (*γ*_*AP*_, *γ*_*PA*_). Grey boxes indicate that the values of the predator and habitat quality variables are hidden, i.e., are not directly observed but have to be inferred by the animal. Different actions are available to the animal depending on its current location. (B) Examples of the evolution of the belief that the habitat is *good*, *β*^*Q*^(*G*). Here, the environment switches from *good* to *bad* after 60s. For most actions, uncertainty will simply increase over time, and for *γ*_*GB*_ = *γ*_*BG*_, will tend to 0.5 (dashed line). If the animal chooses to *feed*, its rate of encounter with *rich* patches (black points) provides information about current habitat quality (solid line). (C) Examples of the evolution of the belief that a predator is *present*, *β*^*D*^(*P*). Here, a predator enters and remains in the environment at 60s. If the animal is in the refuge and selects *rest*, there is no information about the state of predation, and uncertainty increases over time (again to *β*^*D*^(*P*) = 0.5; long dashed line). For most of the other activities, indirect cues *o*_*i*_ (black points) are freely available, which provide some information about the state of predation (short dashed line). For *assess*, both indirect cues *o*_*i*_ and direct cues *o*_*d*_ (red points) are available; the latter provide more reliable information about whether a predator is *present* or *absent* (solid line). Parameters: *γ*_*GB*_ = *γ*_*BG*_ = 0.01, $\rho_{r}^{G}=0.8$, $\rho_{r}^{B}=0.2$; *γ*_*AP*_ = *γ*_*PA*_ = 0.1, $\lambda_{o_{i}}^{-}=\lambda_{o_{d}}^{-}=0.1$, $\lambda_{o_{i}}^{+}=0.5$, $\lambda_{o_{d}}^{+}=0.9$.

We therefore constructed a more detailed example of foraging under predation risk in order to elaborate and amplify these points. Along with making the task recurrent, it includes two structural extensions (shown in Fig 2A). First, we expand the number of environmental variables that might influence the animal’s decisions by additionally allowing the foraging quality of the environment to vary over time. Second, we now model two different locations that the animal may occupy and continuously move between: a *patch* location, in which the animal may forage, and a *refuge* location, where the animal is safe but cannot forage.

More formally, the model is a *partially observable semi-Markov decision process* (POSMDP) which can be described in terms of states, actions, transitions, rewards, observations, and a discount factor.

### States

States are determined by the values of three binary variables: 1) *location* ∈ {*refuge*, *patch*}, where *refuge* affords safety but not food, while *patch* affords foraging but also possible predation; 2) *habitat quality* ∈ {*good*(*G*), *bad*(*B*)}, which determines the current utility of feeding (see below); and 3) *predator* ∈ {*present*(*P*), *absent*(*A*)}, which describes whether there is currently a predator in the vicinity or not. We assume that the animal always knows its location, but that the state of predation and habitat quality are hidden variables whose values need to be inferred based on evidence.

### Actions

As before, choice involves selection of both an action *a* and a duration *τ* for its performance, and we refer to an action-duration pair (*a*, *τ*) as an *activity*.

Possible actions are location-specific. In the *refuge*, the set is $A_{refuge}=\left\{rest,assess,transit\right\}$, where *rest* is a recuperative behaviour; *assess* specifically aims to increase certainty about whether a predator is currently in the environment from a position of relative safety (e.g., sniffing near the entrance of the *refuge*); and *transit* simply means moving to the *patch* location to forage.

In the *patch* location, the set of available actions is $A_{patch}=\left\{feed,assess,freeze,escape\right\}$, where *feed* refers to the ingestion of food; *assess*, as before, is aimed at detecting whether a predator is present, though here from a position of possible danger; *freeze* aims at avoiding predation by decreasing the probability of detection; and *escape* also aims at evading predation but through returning the animal back to the *refuge*.

### Transitions

The patch quality and predator presence are assumed to obey simple semi-Markov dynamics which are independent of the animal’s actions (we capture the consequences of feeding and predation in the rewards; see Discussion). In particular, we assume that (a) the predator transitions from being *absent* (*A*) to *present* (*P*) with transition rate *γ*_*AP*_, and from *present* to *absent* with transition rate *γ*_*PA*_; and similarly, (b) the habitat quality transitions between being *good* (*G*) and *bad* (*B*) according to transition rates (*γ*_*GB*_, *γ*_*BG*_).

In terms of predation risk, a crucial factor is the probability that the animal is detected and subsequently caught if a predator is present, given that the animal is currently engaged in a particular action. One of the more substantial simplifications we make is to assume that being detected inevitably leads to getting caught, but incurs only a fixed, finite, negative reward, rather than having a more extreme sanction. We formulate predation risk directly in terms of a rate of detection, assuming that this is directly mirrored in the predation rate. For *rest* and *assess* in the *refuge* location, we assume that this rate is 0; in the *patch* location, we assume that the detection rate is a function of the current action, written in abbreviated form as *δ*_*a*_, where *a* denotes the current action. We assume that detection rate is lowest when the animal chooses *freeze*, and more generally assume the ordering *δ*_*escape*_ ≥ *δ*_*transit*_ ≥ *δ*_*feed*_ > *δ*_*assess*_ > *δ*_*freeze*_. Note that we assume that the risk of being caught is also lower if the animal chooses *assess*, since *assess* and *freeze* may reasonably be seen as lying on a continuum which trades off the degree of immobility with the amount of information garnered through risk assessment [30].

Habitats which are *good* (*G*) or *bad* (*B*) are defined in terms of the animal’s encounter rate with either *rich* or *poor* patches while feeding. A habitat which is *good* has a relatively high encounter rate $\rho_{r}^{G}$ with *rich* patches and low encounter rate $\rho_{p}^{G}$ with *poor* patches. Conversely, a habitat which is *bad* yields a low encounter rate $\rho_{r}^{B}$ with *rich* patches, and a high encounter rate $\rho_{p}^{B}$ with *poor* patches.

### Rewards

Each action is associated with a reward rate *r*. These are assumed to be negative (indicating an energetic cost) except for *feed* (net positive) and *rest* (zero). For net-negative reward actions, we assume the general ordering *r*_*escape*_ < *r*_*transit*_ ≤ *r*_*assess*_ ≤ *r*_*freeze*_, so that *escape* is assumed to be most costly, while we are generally agnostic about the relative energetic costs of the other actions. For the *feed* action, the reward rate depends on whether the currently-encountered *patch* is *rich* (rate $r_{feed}^{r}$) or *poor* (rate $r_{feed}^{p}$). A large negative reward *r*_*pred*_ ≪ 0 is associated with being detected/caught by a predator (note that this is not a rate), which may be considered a cost of injury (we return to the issue of modelling predation costs in the Discussion).

As in the simple example above, we assume that making a decision incurs a constant decision cost, *c*_*d*_ ≥ 0, which summarizes the computational, and presumably metabolic, costs associated with deliberation about which activity to pursue.

### Observations

The animal’s location is assumed to be directly observed, while the values of the *predator* and *habitat* variables are assumed to be only partially-observable. The animal is therefore required to make inferences about the latter which will depend on both prior knowledge of environment dynamics and observations.

We assume that certain observations are more probable when a predator is *present* rather than *absent*, provided the animal takes appropriate measures to detect them. Two distinct types of cue are assumed. Firstly, we assume that an *indirect*, or ‘passive’, cue *o*_*i*_ is available regardless of the activity in which the animal is engaged (e.g., hearing a rustle in the bushes). This is emitted at a rate $\lambda_{o_{i}}^{+}$ when a predator is present, and at a rate $\lambda_{o_{i}}^{-}$ when a predator is absent. We write ¬*o*_*i*_ for a *non-observation* of *o*_*i*_ when it could potentially have been observed. Secondly, we assume that a *direct*, or ‘active’, cue *o*_*d*_ is additionally available, but only if the animal is engaged in the *assess* activity (e.g., detecting a visual pattern at a particular location in the foliage). This is emitted at a rate $\lambda_{o_{d}}^{+}$ when a predator is present, and at a rate $\lambda_{o_{d}}^{-}$ when a predator is absent. Again, ¬*o*_*d*_ represents the non-observation of *o*_*d*_ when the latter would have been possible. Therefore, the animal may enter into different ‘information states’ regarding the *predator* variable depending on its choice of action, with *assess* providing the most reliable evidence. We assume that neither type of cue is available when the animal is engaged in *rest*. An absence of information is different from information about absence, as in ¬*o*_*i*_ or ¬*o*_*d*_.

Information about habitat quality is assumed to be only available when the animal opts to *feed*. The relevant cue here is the current reward rate $r\in \{r_{feed}^{r},r_{feed}^{p}\}$ experienced while feeding. This will to some degree be informative about (by depending on) habitat quality—*rich* patches are more commonly encountered in a *good* habitat—but will not completely disambiguate the quality of the current habitat, since both types of habitat contain *rich* and *poor* patches.

### Discount factor

The animal is assumed to discount future rewards according to an exponential function with rate *α* ∈ [0, 1] (i.e., a unit reward received after a delay of *τ* seconds is treated as having present value *e*^−*ατ*^, so that a larger value of *α* leads to more rapid discounting). The effect on behaviour of varying the discount rate is not a primary focus of the current work, and it is set to *α* = 0.1 throughout.

### Belief states

In addition to its current location, the animal’s belief about whether a predator is *present* ($\beta_{t}^{D}\left(P\right)$) and whether the habitat is *good* ($\beta_{t}^{Q}\left(G\right)$) are jointly a sufficient basis on which to choose its actions. These collectively form the animal’s ‘belief state’—allowing us to solve the induced belief state semi-Markov decision process.

### Belief state updates

How the animal’s beliefs change over time depends on both prior knowledge of the environment’s dynamics and any pertinent observations made. Since the *predator* and *habitat quality* variables evolve independently, we can consider belief updates for these separately.

In the case where there is no observation (such as when the animal engages in *rest* within the *refuge*), belief updates only depend on the environment dynamics. The two components of the belief state change from time *t* to *t* + *τ* according to

$$\beta_{t+\tau }^{D}\left(P\right)=\frac{\gamma_{AP}}{\gamma_{AP}+\gamma_{PA}}+(\beta_{t}^{D}\left(P\right)-\frac{\gamma_{AP}}{\gamma_{AP}+\gamma_{PA}})e^{-(\gamma_{AP}+\gamma_{PA})\tau },$$ (1)

$$\beta_{t+\tau }^{Q}\left(G\right)=\frac{\gamma_{BG}}{\gamma_{BG}+\gamma_{GB}}+(\beta_{t}^{Q}\left(G\right)-\frac{\gamma_{BG}}{\gamma_{BG}+\gamma_{GB}})e^{-(\gamma_{BG}+\gamma_{GB})\tau }.$$ (2)

For convenience, we consider an approximation to this for *τ* = Δ*t* ≪ 1

$$\beta_{t+\Delta t}^{D}\left(P\right)\approx \beta_{t}^{D}\left(P\right)e^{-\gamma_{PA}\Delta t}+\left(1-\beta_{t}^{D}\left(P\right)\right)\left(1-e^{-\gamma_{AP}\Delta t}\right),$$ (3)

$$\beta_{t+\Delta t}^{Q}\left(G\right)\approx \beta_{t}^{Q}\left(G\right)e^{-\gamma_{GB}\Delta t}+\left(1-\beta_{t}^{Q}\left(G\right)\right)\left(1-e^{-\gamma_{BG}\Delta t}\right).$$ (4)

When additional information is provided by observations, this needs to be combined with prior expectations according to Bayes rule. If the animal is engaged in an activity for which only indirect observations *o*_*i*_ provide information about the state of predation, then from Bayes rule, the updated belief ${\tilde{\beta }}_{t+\Delta t}^{D}\left(P\right)$ having received an instance of *o*_*i*_ in the interval (*t* + Δ*t*) is

$${\tilde{\beta }}_{t+\Delta t}^{D}\left(P\right)\propto P\left(o_{i}|present\right)\beta_{t+\Delta t}^{D}\left(P\right),$$ (5)

where $P\left(o_{i}|present\right)\approx \lambda_{o_{i}}^{+}\Delta t$, and $\beta_{t+\Delta t}^{D}\left(P\right)$ is given by Eq (3). For an omission, ¬*o*_*i*_, the likelihood $P\left(\neg o_{i}|present\right)\approx \lambda_{o_{i}}^{-}\Delta t$ is used instead. If the animal has access to both indirect and direct observations (i.e., when engaging in *assess*), belief updates follow a similar pattern, e.g.,

$${\tilde{\beta }}_{t+\Delta t}^{D}\left(P\right)\propto P\left(o_{d}|present\right)P\left(o_{i}|present\right)\beta_{t+\Delta t}^{D}\left(P\right),$$ (6)

where $P\left(o_{d}|present\right)\approx \lambda_{o_{d}}^{+}\Delta t$, and so forth for the possible combinations of values for *o*_*i*_ and *o*_*d*_.

Relevant information about the quality of the habitat is only available when the animal selects *feed*, and encounters either *rich* or *poor* patches. Thus, the updated belief ${\tilde{\beta }}_{t+\Delta t}^{Q}\left(G\right)$ when encountering a *rich* patch in the interval (*t* + Δ*t*) is

$${\tilde{\beta }}_{t+\Delta t}^{Q}\left(G\right)\propto P\left(rich\;|good\right)\beta_{t+\Delta t}^{Q}\left(G\right),$$ (7)

where $P\left(rich\;|good\right)\approx \rho_{r}^{G}\Delta t$, and $\beta_{t+\Delta t}^{Q}\left(G\right)$ is given by Eq (4). If the encounter is with a *poor* patch, the likelihood $P\left(poor|good\right)\approx \rho_{p}^{G}\Delta t$ is used instead.

Fig 2B and 2C illustrate how beliefs *β*^*Q*^(*G*) and *β*^*D*^(*P*) evolve over time for the various different cases.

### Interruption

When available, interruption is defined as the ability to stop an activity prematurely and make a new decision. That is, given an initial commitment at time *t* to an activity (*a*, *τ*), interruption is the capacity to stop that activity at any intermediate time in the interval (*t*, *t* + *τ*), and make a new decision (at a cost of *c*_*d*_). Interruption can therefore be thought of as an additional, ‘internal’ action; how decisions about this action should be made, as well as issues surrounding its potential cost and mechanisms, are the principal concerns of the following sections.

## Results

While partially-observable problems are generally too large to be solved exactly, the current model is sufficiently simple that we can solve a discretized version of the (actually continuous time and continuous probability) belief state semi-Markov problem for the optimal policy using value iteration. Beliefs *β*^*D*^(*P*) and *β*^*Q*^(*G*) lie in the interval [0, 1] and were discretized at a resolution of Δ*β* = 0.01; time was discretized at intervals of Δ*t* = 1 s; and selections of duration *τ* were from {1, 2, …, 15} seconds. Note that this rather severe discretization makes for some apparent discontinuities in the optimal policy, but the general form of choice remains.

### Basic model behaviour

**Fig 3:**
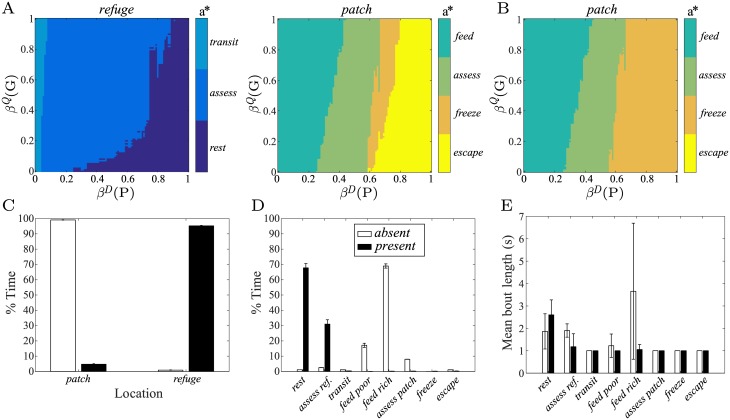
Optimal policies in the absence of a decision cost. (A) Optimal actions a* as a function of current location (left, *refuge*; right, *patch*), and current beliefs that a predator is *present*, *β*^*D*^(*P*), and that the current habitat quality is *good*, *β*^*Q*^(*G*). The discretization of the state space leads to the apparently rough solution. (B) If *escape* does not lead to safety, then *freeze* will always be selected in the *patch* instead. (C–E) The distribution of behaviour shown by the optimal policy depends on whether there is a predator in the environment (black bars) or not (white bars). This includes (C) the proportion of time spent in *refuge* vs. *patch*, and (D) the proportion of time spent in different activities. (E) Even though the policy selects activity durations to be as short as possible (τ* = 1 s), contiguous periods of a given action (‘bouts’) may be longer, reflecting successive choices of the same action. The bars show mean bout lengths measured over 100 instantiations of a 15-minute period; error bars indicate ±1 standard error. Environment parameters as above; reward rates $r_{feed}^{r}=2$, $r_{feed}^{p}=1$, *r*_*rest*_ = 0, *r*_*transit*_ = *r*_*assess*_ = *r*_*freeze*_ = −0.1, *r*_*escape*_ = −1; detection rates *δ*_*feed*_ = *δ*_*transit*_ = *δ*_*escape*_ = 0.05, *δ*_*assess*_ = 0.02, *δ*_*freeze*_ = 0.01, *δ*_*rest*_ = 0; predation punishment *r*_*pred*_ = −100; decision cost *c*_*d*_ = 0.

To examine the basic behaviour of the model, we start by setting the decision cost to zero, *c*_*d*_ = 0. Fig 3A displays the optimal actions a* as a function of the animal’s location, *refuge* (left) or *patch* (right), and belief state {*β*^*D*^(*P*), *β*^*Q*^(*G*)}. In the *refuge*, the animal opts to *transit* to the *patch* only if a predator is unlikely, chooses to *rest* if a predator is likely, and selects *assess* when more uncertain. Choice is modulated by the probability that the habitat quality is *good*: the animal is slightly more likely to tolerate a higher probability that a predator is *present* in order to *transit*, while if the habitat quality is probably *bad*, the animal is increasingly likely to *rest*, in fact even when the probability of a predator is lower than 0.5. Note that even though the utility of *rest* was assumed to be 0, there are conditions under which the animal chooses it anyway. This occurs when the cost associated with *assess* is deemed too high to be worth paying (i.e., when it is highly probable that a predator is *present*, or when the habitat quality is likely to be *bad*). The advantage of *assess* is that it more quickly moves the animal to a state of increased certainty about whether a predator is *present* or not: if the predator is likely *present*, it is better to conserve energy by selecting *rest*; if the predator is likely *absent*, then the sooner the animal chooses to *transit*, the better.

When in the *patch* location, the animal selects *feed* when *β*^*D*^(*P*) is low, *assess* when there is greater uncertainty, and chooses a defensive action ∈ {*freeze*, *escape*} when a predator is more likely than not to be *present*. Again, these tendencies are slightly modulated by the belief *β*^*Q*^(*G*). The decision between *freeze* and *escape* is controlled by a number of factors in the model. Firstly, there is a difference in the cost of performing these actions, as we assumed that *escape* is more costly than *freeze*. Secondly, there is a difference in detectability while performing these actions—it is assumed that the detection rate is higher for *escape* than for *freeze*. Finally, there is the fact—which is the reason why *escape* is selected at all—that a successful *escape* will get the animal back to safety, while *freeze* leaves the animal in the *patch* location. Unsurprisingly, if *escape* is rendered ineffectual, in the sense that its performance also leaves the animal in the *patch*, then *freeze* is always preferred (Fig 3B), consistent with changes in defensive pattern observed in rats and mice when flight is not possible [30, 31].

As in the simple example considered above, when there is no decision cost it is always optimal for the animal to choose *τ* to be as short as possible (see ‘Supporting information’, S1 Fig). This is because there is no cost to doing so, while, in the absence of an interrupt, there is a potential cost of committing to longer durations (*viz.*, not being able to change course of action if observations indicate a change in the environment). *τ* becomes relevant when we consider a nonzero decision cost below.

In Fig 3C–3E, we summarize aspects of behaviour when the optimal policy is repeatedly exposed to an environment where a predator is either *absent* (white bars) or *present* (black bars). Unsurprisingly, when a predator is *present*, the animal spends most of its time in the *refuge* (Fig 3C) engaged in either *rest* or *assess* activity (Fig 3D), whereas it spends most of its time feeding in the *patch* when there is no predator. The adaptiveness of these behaviours is evident, and qualitatively similar reconfigurations of activity patterns in response to predator presence/absence are observed in laboratory-based ethological studies (e.g., [30]). Fig 3E makes the further point that even if the shortest duration *τ* is always selected, this doesn’t mean that ‘bouts’ of behaviour, defined as continuous periods of performing a single action, will always be of minimal duration. Instead, an external observer would sometimes measure longer behavioural bouts, particularly in the case of *rest* and *feed* activities.

### The price of responsiveness: Decision cost, without interrupt

**Fig 4:**
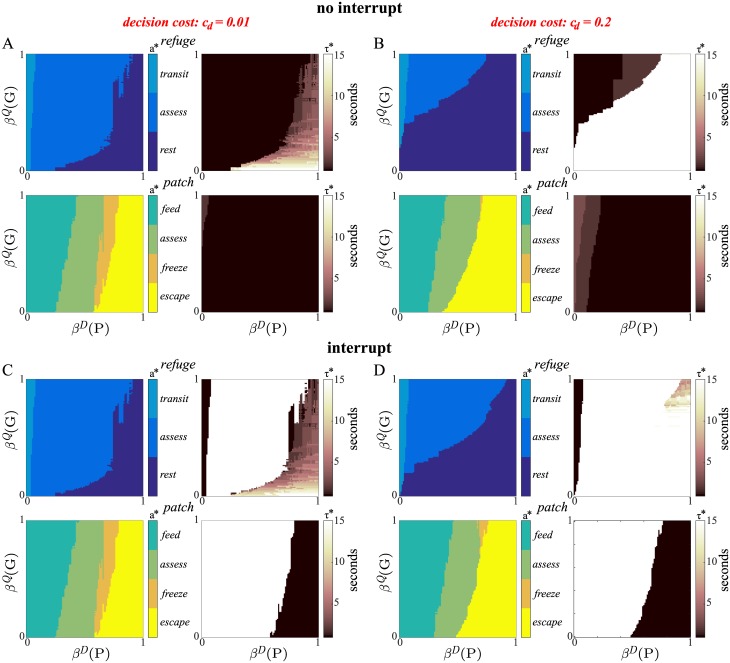
Non-zero decision costs encourage temporally-extended activity when interruption is possible. In each case, there are four plots: the optimal choices of action a* (left plots) and duration τ* (right plots) are shown as a function of location (*refuge*, upper; *patch*, lower) and belief {*β*^*D*^(*P*), *β*^*Q*^(*G*)}. (A;B) Optimal non-interruptible policies for decision costs (A) *c*_*d*_ = 0.01 and (B) *c*_*d*_ = 0.2. (C;D) Optimal interruptible policies for the same decision costs: (C) *c*_*d*_ = 0.01 and (D) *c*_*d*_ = 0.2. Parameters otherwise set as above.

As the decision cost rises from zero (*c*_*d*_ > 0), optimal behaviour changes. Fig 4A shows the optimal policy for decision cost *c*_*d*_ = 0.01, now displaying both optimal actions a* (left panels) in each location and corresponding optimal durations τ* (right panels). Optimal actions a* are essentially identical to the *c*_*d*_ = 0 case above (cf. Fig 3A), and optimal durations τ* are generally selected to be as short as possible. The exception engendered by this rather minimal decision cost comes in the case of *rest*, where longer durations are observed, particularly when the habitat is likely to be *bad*. These reflect the dynamics of the environment: if habitat quality is currently likely to be *bad*, and conditions change relatively slowly, then conditions are likely to remain *bad* in the near future. Thus, rather than making another (costly) decision to *rest* at that future time, the animal can safely commit to *rest* for a longer period.

By contrast, all other actions are associated with short durations. As in the previous case, this is sensible considering the consequences of doing otherwise. For example, when in the *refuge*, an animal that commits to *assess* for an extended duration may thereby forego time that could be better spent foraging or resting; in the *patch*, the same commitment risks foregoing the opportunity to *feed* or respond defensively (*freeze*/*escape*).

If decisions are made even more expensive, e.g. *c*_*d*_ = 0.2, further changes in policy are observed (Fig 4B). In the *refuge*, *rest* becomes the most prominent action, with a duration that is uniformly chosen to be the longest possible. This minimizes decision costs when the environment is determined to be unfavourable. Note that under the assumed environment dynamics, beliefs *β*^*D*^(*P*) and *β*^*Q*^(*G*) will both move towards 0.5 once *rest* is initiated (recall that no observation is available during this activity), and yet the belief state (0.5, 0.5) yields the selection of *rest* under this policy. In other words, once this policy initiates *rest*, it will never do anything else, since the potential benefits of doing anything else are outweighed by the costs (i.e., any other option must have an average reward < 0, since *rest* has net reward 0). Of course, in reality, various factors would militate against this, including the stochasticity of action choice and progressive starvation (see Discussion). In the *patch*, it is notable that *freeze* has all but disappeared from the behavioural repertoire, replaced by *escape*. This is because its benefits—lower detectability and the avoidance of unnecessary excursions back to the *refuge*— are now outweighed by the burden of greater future decision costs incurred in the *patch*. Some subtle increases in τ* are discernible for *feed* at low levels of *β*^*D*^(*P*); but overall, variation in τ* remains limited.

### The benefit of interruptibility: Decision cost, with interrupt

What happens if we now allow the animal to interrupt activities prior to their completion? The animal’s optimal policy only recommends the activity (a*, τ*) in initial belief state {*β*^*D*^(*P*), *β*^*Q*^(*G*)} which is best *in expectation* over possible future belief trajectories. It is therefore perfectly possible that, having chosen optimally with respect to the initial belief state, experience sends the animal on a particular trajectory where it reaches a belief state in which an alternative activity would be preferable. Described at the level of a meta-decision, interruption should occur exactly when the benefit of interrupting an activity is greater than that of continuing it (cf. [15]).

Fig 4C shows the optimal interruptible policy for the same *c*_*d*_ = 0.01 case as before. The clear difference is that for actions *assess*, *feed*, and *freeze*, it is optimal to set the duration to be as *long* as possible: τ* = *τ*_max_. Equipped with the (free) option of interrupting itself at any time, the animal can minimize its decision costs by provisionally committing to long activity durations, only interrupting and making another decision when it really needs to.

The exceptions are *transit* and *escape*. For *transit* (i.e., moving from *refuge* to *patch*), choosing a longer duration never makes any sense—it would only increase the associated energetic cost and predation risk—and there is no advantage to interrupting this activity in the model. The same reasoning applies to *escape*. Since beliefs evolve in a predictable manner for *rest*, there is never a reason to interrupt this activity—the duration is calibrated in the same manner as in the non-interruptible case.

Fig 4D displays the optimal interruptible policy for the more expensive, *c*_*d*_ = 0.2, case. *Rest* begins to occupy greater regions of belief space when in the *refuge*. This is similar to the trend in the non-interruptible case (cf. Fig 4B), albeit to a lesser extent. The interruptible policy does not get ‘stuck’ permanently selecting *rest*, but will rather select *assess* when uncertainty is greatest (i.e., at (0.5, 0.5)), and at many other points in this region. Note also that in contrast to the uniform choice of the longest duration for *rest* in the non-interruptible case, there is still some gradation in choice of *τ* in the interruptible policy (Fig 4D, upper right panel): when it is strongly believed that habitat quality is currently *good*, it is better to choose shorter durations of *rest* to be able to take advantage of predator-free foraging conditions in the near future (cf. Fig 4A and 4C).

Equipped with the capacity to interrupt itself, an animal should perform at least as well as when lacking this capacity (cf. [15], Theorem 2). At worst, decisions could be taken at maximum frequency, and the same decision costs incurred as in the non-interruptible case. We expect the interruptible case to do better than this, however: interruption should allow the animal to commit to extended activity flexibly, and so decrease the cost of unnecessary decisions—just as in the simplified example we first considered.

**Fig 5:**
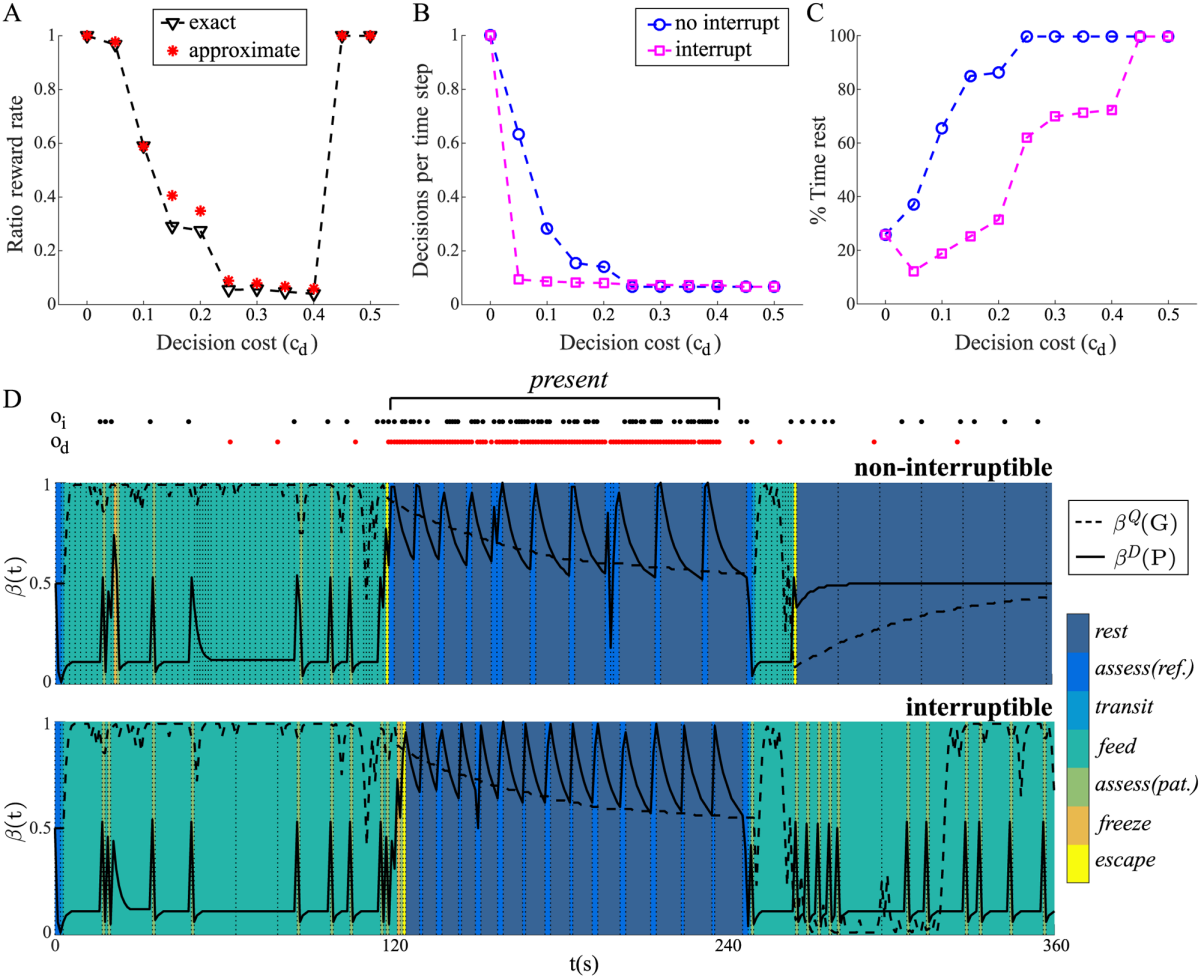
The ability to interrupt on-going behaviour confers a reward advantage. (A) Ratio of average total amount of reward per trial (non-interrupt/interrupt) for different decision costs. This is shown both for the exact interruptible policy (black triangles, dashed line), and the approximate interruptible policy (red asterisks) which is based on a linear approximation (as detailed in the Section ‘A cheap interruption mechanism’ below). For each decision cost, optimal non-interruptible and interruptible policies performed 1000 trials. (B) Average number of decisions per time step and (C) average percentage time spent in *rest* for the optimal non-interrupible (blue circles) and interruptible (magenta squares) policies. (D) Behaviour and beliefs of the optimal non-interruptible (upper) and interruptible (lower) policy for a particular trial when *c*_*d*_ = 0.1. Time points at which decisions are made are indicated by vertical dashed lines. Model parameters otherwise set as above.

As in that example, we compared performance in a simulated experiment. Here, behaviour is measured over a 6-minute period in which a predator is initially *absent* (2 min), then *present* (2 min), then *absent* again (2 min); habitat quality is allowed to fluctuate randomly. We ran this experiment 1000 times for different settings of the decision cost *c*_*d*_, ensuring that conditions were exactly matched between policies. We measured the resulting rewards averaged over both episodes and experiments. Fig 5A (inverted triangles; ‘exact’) plots the reward rate achieved by the non-interruptible policy as a fraction of that achieved by the interruptible policy (cf. Fig 1G). As seen before, when *c*_*d*_ = 0, this fraction is 1, reflecting the fact that the optimal strategy here is to make decisions as often as possible, since there is no penalty to doing so. However, as decisions become increasingly expensive (i.e., *c*_*d*_ becomes larger), the fractional utility rate decreases, reflecting the fact that the interruptible policy is achieving higher rewards. Beyond a certain level of decision cost (here, *c*_*d*_ > 0.4), the fraction reverts to 1.

Again, the reward advantage comes from the reduction in the frequency of decisions that is possible when the animal has the capacity to interrupt (Fig 5B). Reversion of the fractional reward rate to 1 in this case corresponds to the point at which simply spending all of its time engaged in *rest* is the optimal course of action for the animal, regardless of the capacity to interrupt. If we explicitly compare the average time during the experiment spent at *rest* for non-interruptible and interruptible policies, both are eventually driven to exclusive choice of this action as decisions become more expensive. However, this occurs for lower values of *c*_*d*_ when interruption is unavailable (Fig 5C).

Fig 5D compares the behaviour and beliefs of the non-interruptible (upper) and interruptible (lower) policies for a particular run of the experiment at an intermediate decision cost, *c*_*d*_ = 0.1. This clearly illustrates the fact that the non-interruptible policy makes decisions at a much higher frequency (vertical dashed lines), but also highlights differences in choices. Most notably, in both cases the animal returns to the *patch* after the predator has been removed from the environment, but when faced with observations that may indicate a potential threat, their behaviour differs. In the non-interruptible case, the animal opts to escape to the *refuge* and engage in *rest*, since the costs of foraging are deemed too high when a predator is thought likely and habitat quality is low (Fig 5D, upper). By contrast, in the interruptable case, the animal opts to *assess* whether there is a real threat, and continues to *feed* when it transpires that no predator is around—it is sufficiently flexible in its behaviour for it to be worthwhile to continue in the *patch* and intermix periods of both feeding and assessment (Fig 5D, lower). Note also the shorter bouts of *rest* in the interruptible case, leading to a greater frequency of *assess* and so, at least in this case, a marginally earlier return to the *patch*.

### A cheap interruption mechanism

Interruption is evidently useful; however, we have not considered the costs of the calculations associated with this operation. Since interruption can be thought of as another layer of decision-making, we might just have increased the computational burden. One answer is to conceive of a cheap and ‘light-weight’ interruption process.

The points at which interruption should be triggered effectively form a boundary on belief space. We might therefore consider that at the outset of an activity, one could ‘construct’ such a boundary, or *threshold*, and then have interruption occur whenever this threshold is breached. Belief-monitoring would still be required to recognize if and when the animal enters the termination set of beliefs. This still implies a decision—whether or not this threshold has been reached—but of a particularly simple kind.

**Fig 6:**
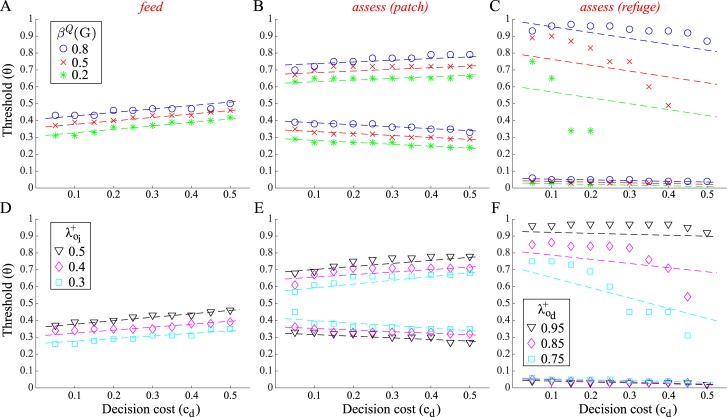
Optimal and approximate interruption thresholds. (A–C) Optimal thresholds *θ* (symbols) for degree of belief *β*^*D*^(*P*) as a function of decision cost *c*_*d*_ and (fixed) belief level *β*^*Q*^(*G*). These are shown for (A) *feed*, (B) *assess* (in *patch*), and (C) *assess* (in *refuge*). The lines show a linear approximation to the thresholds. (D–F) Optimal and approximate thresholds for the same activities as a function of *c*_*d*_ and cue reliability (i.e., higher or lower true positive rate for indirect observations, $\lambda_{o_{i}}^{+}$, or direct observations, $\lambda_{o_{d}}^{+}$); *β*^*Q*^(*G*) = 0.5. Unless otherwise indicated, the default reliabilities were $\lambda_{o_{i}}^{-}=0.1$, $\lambda_{o_{i}}^{+}=0.5$, $\lambda_{o_{d}}^{-}=0.1$, $\lambda_{o_{d}}=0.9$. Other parameters as previous.

Fig 6A–6C (symbols) plot the optimal interruption thresholds *θ*, i.e., levels of belief *β*^*D*^(*P*) at which activities should be interrupted, as a function of decision cost *c*_*d*_ and three fixed levels of belief *β*^*Q*^(*G*). The thresholds are plotted for *feed* and for *assess* ∈ {*patch*, *refuge*}, which are activities that show a robust increase in duration with nonzero decision costs (cf. Fig 4C and 4D above); since *freeze* tends to be disfavoured at higher decision costs (cf. Fig 4D), data points are much more sparse, and so we do not consider it here. Note that *feed* only has an upper boundary on *β*^*D*^(*P*), while *assess* has both upper and lower boundaries. Also, for some combinations of *c*_*d*_ and *β*^*Q*^(*G*), the set of beliefs {*β*^*D*^(*P*)} for which *assess* in the *refuge* location is optimal (i.e., the ‘initiation set’) is empty, and so interruption thresholds are omitted in these cases (e.g., for *β*^*Q*^(*G*) = 0.2, *c*_*d*_ > 0.2; Fig 6C).

As one might expect from the example optimal policies seen previously (cf. Fig 4C and 4D), the interruption threshold for *feed* increases for higher *β*^*Q*^(*G*), i.e., as the payoff for feeding increases (Fig 6A). With *assess* in the *patch* location, the upper and lower thresholds move upwards as *β*^*Q*^(*G*) increases, respectively indicating a greater willingness to interrupt and initiate feeding (lower boundary), and a greater reluctance to trigger defensive behaviour (upper boundary) (Fig 6B). For *assess* in the *refuge*, the upper boundary moves upwards as *β*^*Q*^(*G*) increases, indicating greater reluctance to interrupt and initiate *rest* when habitat quality is likely to be *good*; the lower boundary shows less variation—the animal requires *β*^*D*^(*P*) to be very low to interrupt and initiate *transit*, regardless of *β*^*Q*^(*G*) (Fig 6C).

The thresholds *θ* for the first two cases are well approximated by linear functions of *β*^*Q*^(*G*) and *c*_*d*_, while this is less true of *assess* in the *refuge* location (Fig 6A–6C, dashed lines). We can nevertheless ask how well, in comparison to the optimal policy, an animal will do when selecting interruption thresholds according to the linear function which most closely approximates the optimal thresholds. Fig 5A (red asterisks) shows that this simple, approximate way of setting thresholds leads to benefits that are extremely close to that of the exact case.

It is also informative to examine how interruption thresholds change as a function of cue reliability. Fig 6D–6F show these as a function of decision cost *c*_*d*_ and true positive rates for either indirect cues, $\lambda_{o_{i}}^{+}$ (Fig 6D), or direct cues, $\lambda_{o_{d}}^{+}$ (Fig 6E and 6F), while *β*^*Q*^(*G*) is kept constant. Reducing the true positive rate in either case means less reliable information about the predator.

For *feed*, thresholds decrease with less reliable indirect cues, consistent with greater caution—the animal is increasingly prepared to interrupt feeding in order to *assess* and gain better information (Fig 6D).

For *assess* in the *patch* location, upper thresholds similarly decrease with less reliable direct cues, reflecting a greater willingness to trigger interruption and switch to defensive behaviour (Fig 6E, upper). That lower thresholds actually increase with less reliable cues indicates a greater willingness to interrupt and initiate *feed* in spite of having less reliable predation cues; this might seem the exact opposite of more cautious behaviour. The reason is that gathering information via *assess* decreases in marginal value for unreliable cues, making *feed* an increasingly attractive alternative. Note that the relative reliabilities of direct and indirect cues are important in this trade off, since *feed* still provides some information via the latter class of cues.

Finally, thresholds for *assess* in the *refuge* location follow a qualitatively similar pattern (Fig 6F). With less reliable cues, the animal is quicker to interrupt its behaviour and initiate *rest*, thereby conserving its energy; with more reliable cues, the animal is more conservative in making this transition (Fig 6F, upper thresholds). The difference in thresholds is less pronounced in the downwards direction, so that a relatively high degree of certainty regarding predator absence is required before initiating *transit* in all cases.

## Discussion

We used the simple example of foraging under predation risk to explore the possible advantages of being able to interrupt on-going behaviours. We showed that this allows animals to make provisional commitments to courses of behaviour rather than having either to check obsessively or just not to exploit available resources at all. This had measurable benefits in terms of efficiency and effectiveness. We also showed that it is not necessary to solve a complex decision problem to choose whether to interrupt, but rather that a simple, cheaply-parameterized, approximate, threshold-based policy can perform almost as well as the optimal policy across a variety of parameter settings.

In our model, decision cost was summarized by a simple scalar value. However it may in reality comprise separate components, including an intrinsic (e.g., metabolic) cost of performing the computation, and an opportunity cost, which summarizes what could have been obtained by employing the engaged resource (time, computation) otherwise [32–34]. The cost we considered, *c*_*d*_, is a version of the former, and could arise from steps of expansion and calculation in a decision tree used for planning. The opportunity cost of time arises from discounting—the fact that taking time to think postpones *future* rewards, making them less valuable (an issue more extensively explored in the case of long-run average reward; cf. [35]). The opportunity cost of the use of other cognitive resources for deliberation, such as working memory, are starting to be considered and quantified [36–39]. Model-free planning [40, 41] is likely to impose far smaller cognitive costs than the sort of model-based planning that we have so far been considering. However, at the very least, there will still be costs associated with task switching [42].

The activities (*a*, *τ*) that formed the objects of choice in the current work may be recognised as a simple form of *option* [15]—a policy for taking actions over an extended period of time—which has formed one basis (amongst many, cf. [43]) for exploring the issue of temporal abstraction in reinforcement learning. The benefit of being able to interrupt an option before it would otherwise terminate was highlighted in the initial options paper by Sutton and colleagues [15] (the authors also cite previous work by Kaelbling [44]), but issues surrounding decision cost, plausible mechanisms for interruption, and partial observability were not considered there. More recent work by Precup and colleagues [45], who introduce an ‘option-critic architecture’ in the context of discovering and learning options, explicitly considers the idea that a form of switching cost could encourage commitment to option execution.

We showed that changing the informativeness of cues about the predator had consequences such as increasing the propensity to feed and altering interruption thresholds. The effects of such changes on the observed temporal structure of behaviour are subtle, since the speed with which the belief about the presence of the predator changes will also change. In the case we simulated, observations remained sufficiently informative that the latter effect had little impact, but it would be interesting to examine more systematically how thresholds and speeds interact in determining when interruption occurs. Since this would require some significant adjustments to the current model, we leave exploration of this subtlety for future work.

The trade off between energetic gain and predation risk is a central topic of behavioural ecology [27, 46] and has been the subject of extensive previous theoretical work, though principally at more ‘molar’ levels of analysis than our approach here [28, 29, 47, 48]. While our model of foraging under predation risk was loosely inspired by ethological and ethoexperimental studies of rodent behaviour in such settings [30, 49–51], a more realistic model would extend this in a number of ways.

A first extension concerns the model of predation. Most notably, in our detailed model, getting caught by the predator was associated with a large negative cost (as from a severe injury) rather than an outright termination of the decision process (as from extermination). If the cost of injury is sufficient, then the difference becomes rather moot; however, a more realistic model involving procreation and death from natural and unnatural causes would be most interesting. Second, we made the assumption that when a predator is *present*, the animal has a constant probability of being detected and harmed—indeed, we made no distinction between detection and being caught, and have not otherwise separated out the ‘subcomponents’ of predation risk [27]. Third, we assumed that the rate at which a predator enters and leaves the environment is constant, whereas one might expect that the predator would be more likely to remain in the environment if it has detected prey. Finally, since the model is non-spatial, it cannot address important factors such as differences in time to reach safety from different locations, and associated variation in the distance an animal will tolerate from a simulated predator before initiating flight (‘flight-initiation distance’ in the light of predatory imminence [51–53]).

Further unmodelled complexity arises through the behavioural sophistication that animals display both in assessing predation risk and in responding to the presence of a predator. These behaviours have been extensively studied in wild and laboratory rats [49, 51], including investigations of approach-avoidance conflict [54]. Predatory risk assessment alternates between cautious forays into a potentially dangerous area and rapid retreat to safety, if available. If escape or concealment is not possible, the animal instead alternates between freezing and scanning with the head and vibrissae. When actually confronted with a predator, a rat will variously respond by fleeing, freezing, or attacking, depending on the nature of the current environment—particularly whether a place of relative safety, or refuge, is available—and the intensity of the perceived threat, or ‘defensive distance’ [55]. Capturing the latter concept would require a richer spatial and perhaps temporal model.

There were also marked simplifications concerning the benefits of foraging, both in terms of the agent and the environment. In terms of the former, we did not capture the possibility of running out of resources. Thus, for instance, it would have been possible for the agent to stay in *rest* in perpetuity (as indeed would seem optimal for expensive decision-making and no interruption; Fig 4B). In reality, as threats to homeostatic integrity loom, we can expect animals to exhibit more risk-seeking behaviour [56]. This would emerge in the current formulation given a more realistic characterization of the utilities [57]. In terms of the environment, a key simplification is to ignore resource depletion by the agent, and the existence of multiple patches. Then, critical concerns in foraging theory such as the marginal value theorem [58] would be important, and the rate of prey encounter or capture might also contribute to interruption.

While we primarily focused on the computational and algorithmic aspects of interruption, it is of clear interest to relate the current work to neural substrates. The present theory of interruption can be seen as a mesoscopic bridge between the macroscopic view of the neuromodulator norepinephrine (NE) suggested by the reversal experiments of Devauges and Sara [20], in which it reports changes to the whole rules of the environment [25], and the microscopic view of Bouret and Sara [21], and Dayan and Yu’s [26] interpretation of [22], in which it reports the current level of uncertainty about the ongoing belief state in a single perceptual inference problem, triggering interruption on reaching a pre-defined threshold, allowing switching to a better hypothesis [59]. This was proposed as part of the approximate strategy of provisionally committing to a single hypothesis, but keeping track of how this might be erroneous. At all levels, the common theme is the question of whether to interrupt a default state, whether that be a default belief about the current state of the world, as in [25, 26], or a default program of activity, as in the current case. Closer examination of recordings from noradrenergic neurons and associated circuitry during naturalistic behaviour (including foraging tasks) for evidence of the sort of multilevel dynamics predicted by these three accounts would therefore be merited. NE activity has also be associated with arousal [60]; the relationship between this concept and that of interrupts is the subject of on-going theoretical work. Its association with other functions such as learning [61, 62] and exploration [23, 63, 64] are arguably further removed. Possible divisions of labour and interactions between cortical and subcortical systems in this context are also of interest [65].

One can speculate about the relationship between the putative function of NE as an interrupt and its association with stress-induced anxiety [66, 67]. In environments with a high proportion of unpredictable events, or indeed an environment that is either frankly aversive or believed to contain possible sources of threat, the interrupt mechanism is likely to be frequently engaged, whether received stimuli reflect real threats or not. This state of high interruptibility, or distractibility—reminiscent in some respects of ‘hypervigilance’—is plausibly associated with higher costs, both in terms of time and energy, and would be manifest in our own model in a greater frequency of costly deliberations. In the limit of an extremely inconstant environment, interruptibility loses any net benefit; however, whether this happens before the costs are such that the animal will refuse to engage with the environment at all depends on the details of the cost structure.

All these forms of interrupt are likely to be distinct from the motor interrupt that plays a critical role in tasks such as the stop signal reaction time task [68], or the ‘hold your horses’ interrupt [69, 70] that has been suggested to suppress a prepotent action temporarily to allow time for a correct choice to be made. The former may also be associated with the form of cognitive state change associated with the phasic NE signal [21, 26], but this would be a distinct consequence of the same underlying detection. Indeed, the neural substrates for these others forms of inhibition are notably different, implicating regions of the superior colliculus and basal ganglia, respectively.

Although we focused on the benefits of interruption and its possible mechanisms in the context of foraging, similar considerations are expected to be more generally applicable. One area of particular interest is decisions about ‘internal’ rather than external behaviour (i.e., meta-cognition). Indeed, the interpretation of NE we mentioned above as a signal for interrupting a likely incorrect ongoing belief is an example of this [26]. The strategy of provisional commitment could be particularly beneficial when there are such large numbers of potential hypotheses that they cannot simultaneously be entertained.

As another example, consider the problem of trying to decide whether to perform a particular action by considering its possible future consequences (i.e., by model-based planning; [40]). Ultimately, this will be intractable due to the myriad possibilities, and the challenge of determining the uncertainties and utilities of each. However, some degree of planning should be useful, until the fog of uncertainties about the future and the complexity of calculating it overwhelm the utility of attempting to do so. This question has also been of great interest in artificial intelligence [1, 71, 72].

One could try to determine how deep to plan before planning—planning to plan—but doing so optimally presents an even more formidable computational challenge. A more realistic option would be to commit to the planning process provisionally, while monitoring its progress (e.g., the extent to which one’s uncertainty about the value of the action decreases with planning depth), and to interrupt this process when further planning appears unjustifiable (see, e.g., [73]). In such cases of diminishing marginal returns, interruption according to a relatively simple threshold rule may apply, similar to the logic of the marginal value theorem in foraging theory [32, 58]. Consideration of how long to run an algorithm is a central concern of work on anytime algorithms (or ‘interruptible’ algorithms), i.e., algorithms which are always guaranteed to return a valid solution but where the solution quality typically improves with time [6, 74, 75]. It is pressing to consider these insights in the light of what we know about the neural substrates of interruption.

## Supporting information

S1 Fig Optimal policies in the absence of a decision cost. When there is no decision cost, it is always optimal to choose *τ* to be as short as possible.
(EPS)

## References

[1] Russell SJ, Norvig P. Artificial intelligence: A modern approach, 3rd edition Pearson; 2009.

[2] Broadbent DE. Perception and communication. New York: Pergamon Press; 1958.

[3] Kahneman D. Attention and effort. New York: Prentice Hall; 1973.

[4] Pashler H. The psychology of attention. Cambridge, MA: MIT Press; 1996.

[5] Simon HA. Models of bounded rationality, volume 1. Cambridge, MA: MIT Press; 1982.

[6] Horvitz EJ. Reasoning about beliefs and actions under computational resource constraints. In: Proceedings of the Third Workshop on Uncertainty in Artificial Intelligence. Mountain View, CA: AAAI Press; 1987. p. 429–444.

[7] Houston A, Sumida B. A positive feedback model for switching between two activities. Animal Behaviour. 1985;33(1):315–325. doi: 10.1016/S0003-3472(85)80145-7

[8] Marshall JA, Favreau-Peigne A, Fromhage L, Mcnamara JM, Meah LF, Houston AI. Cross inhibition improves activity selection when switching incurs time costs. Current Zoology. 2015;61(2):242–250. doi: 10.1093/czoolo/61.2.242

[9] Monsell S. Task switching. Trends in Cognitive Sciences. 2003;7(3):134–140. doi: 10.1016/S1364-6613(03)00028-7 12639695 10.1016/s1364-6613(03)00028-7

[10] Brooks R. A robust layered control system for a mobile robot. IEEE Journal of Robotics and Automation. 1986;2(1):14–23. doi: 10.1109/JRA.1986.1087032

[11] Agre PE, Chapman D. Pengi: An implementation of a theory of activity. In: Proceedings of the Sixth National Conference on Artificial Intelligence. Menlo Park, California: AAAI Press; 1987. p. 268–272.

[12] Maes P. Designing autonomous agents: Theory and practice from biology to engineering and back. Cambridge, MA: MIT press; 1990.

[13] Wilson SW. The animat path to AI. In: Meyer JA, Wilson SW, editors. From Animals to Animats: Proceedings of the First International Conference on Simulation of Adaptive Behavior. Cambridge, MA: MIT Press; 1991. p. 15–22.

[14] Sacerdoti ED. Planning in a hierarchy of abstraction spaces. Artificial Intelligence. 1974;5(2):115–135. doi: 10.1016/0004-3702(74)90026-5

[15] Sutton RS, Precup D, Singh S. Between MDPs and semi-MDPs: A framework for temporal abstraction in reinforcement learning. Artificial Intelligence. 1999;112(1-2):181–211. doi: 10.1016/S0004-3702(99)00052-1

[16] Wilkins DE. Recovering from execution errors in SIPE. Computational Intelligence. 1985;1(1):33–45. doi: 10.1111/j.1467-8640.1985.tb00057.x

[17] McDermott D. Planning and acting. Cognitive Science. 1978;2(2):71–100. doi: 10.1207/s15516709cog0202_1

[18] Hoffmann J, Brafman R. Contingent planning via heuristic forward search with implicit belief states. In: Biundo S, Myers K, Rajan K, editors. Proceedings of the Fifteenth International Conference on Automated Planning and Scheduling. vol. 2005. AAAI Press; 2005. p. 71–80.

[19] Silberschatz A, Galvin PB, Gagne G. Operating systems concepts. John Wiley & Sons; 2013.

[20] Devauges V, Sara SJ. Activation of the noradrenergic system facilitates an attentional shift in the rat. Behavioural Brain Research. 1990;39(1):19–28. doi: 10.1016/0166-4328(90)90118-X 2167690 10.1016/0166-4328(90)90118-x

[21] Bouret S, Sara SJ. Network reset: A simplified overarching theory of locus coeruleus noradrenaline function. Trends in Neurosciences. 2005;28(11):574–582. doi: 10.1016/j.tins.2005.09.002 16165227 10.1016/j.tins.2005.09.002

[22] Clayton E, Rajkowski J, Cohen JD, Aston-Jones G. Phasic activation of monkey locus ceruleus neurons by simple decisions in a forced-choice task. The Journal of Neuroscience. 2004;24:9914–9920. doi: 10.1523/JNEUROSCI.2446-04.2004 15525776 10.1523/JNEUROSCI.2446-04.2004 PMC6730226

[23] Aston-Jones G, Cohen JD. An integrative theory of locus coeruleus-norepinephrine function: Adaptive gain and optimal performance. Annual Reviews Neuroscience. 2005;28:403–450. doi: 10.1146/annurev.neuro.28.061604.135709 10.1146/annurev.neuro.28.061604.135709 16022602

[24] Sara SJ. The locus coeruleus and noradrenergic modulation of cognition. Nature Reviews Neuroscience. 2009;10(3):211–223. doi: 10.1038/nrn2573 19190638 10.1038/nrn2573

[25] Yu AJ, Dayan P. Uncertainty, neuromodulation, and attention. Neuron. 2005;46:681–692. doi: 10.1016/j.neuron.2005.04.026 15944135 10.1016/j.neuron.2005.04.026

[26] Dayan P, Yu AJ. Phasic norepinephrine: A neural interrupt signal for unexpected events. Network: Computation in Neural Systems. 2006;17(4):335–350. doi: 10.1080/09548980601004024 10.1080/09548980601004024 17162459

[27] Lima SL, Dill LM. Behavioral decsions made under the risk of predation: A review and prospectus. Canadian Journal of Zoology. 1990;68:619–640. doi: 10.1139/z90-092

[28] Houston AI, McNamara JM. Models of adaptive behaviour. Cambridge: Cambridge University Press; 1999.

[29] Houston AI, McNamara JM, Hutchinson JMC. General results concerning the trade-off between gaining energy and avoiding predation. Philosophical Transactions: Biological Sciences. 1993;341(1298):375–397. doi: 10.1098/rstb.1993.0123

[30] Blanchard DC, Blanchard RJ. Ethoexperimental approaches to the biology of emotion. Annual Review of Psychology. 1988;39:43–68. doi: 10.1146/annurev.ps.39.020188.000355 2894198 10.1146/annurev.ps.39.020188.000355

[31] Vale R, Evans DA, Branco T. Rapid spatial learning controls instinctive defensive behavior in mice. Current Biology. 2017;27:1–8. doi: 10.1016/j.cub.2017.03.031 28416117 10.1016/j.cub.2017.03.031 PMC5434248

[32] Boureau YL, Sokol-Hessner P, Daw ND. Deciding how to decide: Self-control and meta-decision making. Trends in Cognitive Sciences. 2015;19(11):700–710. doi: 10.1016/j.tics.2015.08.013 26483151 10.1016/j.tics.2015.08.013

[33] Kurzban R, Duckworth A, Kable JW, Myers J. An opportunity cost model of subjective effort and task performance. Behavioral and Brain Sciences. 2013;36(6):661–679. doi: 10.1017/S0140525X12003196 24304775 10.1017/S0140525X12003196 PMC3856320

[34] Shenhav A, Musslick S, Lieder F, Kool W, Griffiths TL, Cohen JD, et al Toward a rational and mechanistic account of mental effort. Annual Review of Neuroscience. 2017;40:99–124. doi: 10.1146/annurev-neuro-072116-031526 28375769 10.1146/annurev-neuro-072116-031526

[35] Niv Y, Daw ND, Joel D, Dayan P. Tonic dopamine: Opportunity costs and the control of response vigor. Psychopharmacology. 2007;191(3):507–520. doi: 10.1007/s00213-006-0502-4 17031711 10.1007/s00213-006-0502-4

[36] Kool W, McGuire JT, Rosen ZB, Botvinick MM. Decision making and the avoidance of cognitive demand. Journal of Experimental Psychology: General. 2010;139:665–682. 20853993 10.1037/a0020198 PMC2970648

[37] Keramati M, Dezfouli A, Piray P. Speed/accuracy trade-off between the habitual and the goal-directed processes. PLoS Computational Biology. 2011;7:e1002055 doi: 10.1371/journal.pcbi.1002055 21637741 10.1371/journal.pcbi.1002055 PMC3102758

[38] Pezzulo G, Rigoli F, Chersi F. The mixed instrumental controller: Using value of information to combine habitual choice and mental simulation. Frontiers in Psychology. 2013;4:92 doi: 10.3389/fpsyg.2013.00092 23459512 10.3389/fpsyg.2013.00092 PMC3586710

[39] Keramati M, Smittenaar P, Dolan RJ, Dayan P. Adaptive integration of habits into depth-limited planning defines a habitual-goal-directed spectrum. Proceedings of the National Academy of Sciences. 2016;113(45):12868–12873. doi: 10.1073/pnas.1609094113 10.1073/pnas.1609094113 PMC5111694 27791110

[40] Sutton RS, Barto AG. Reinforcement learning: An introduction. Cambridge, MA: MIT Press; 1998.

[41] Daw ND, Niv Y, Dayan P. Uncertainty-based competition between prefrontal and dorsolateral striatal systems for behavioral control. Nature Neuroscience. 2005;8(12):1704–11. doi: 10.1038/nn1560 16286932 10.1038/nn1560

[42] Rogers RD, Monsell S. Costs of a predictible switch between simple cognitive tasks. Journal of Experimental Psychology: General. 1995;124(2):207 doi: 10.1037/0096-3445.124.2.207

[43] Barto AG, Mahadevan S. Recent advances in hierarchical reinforcement learning. Discrete Event Dynamic Systems: Theory and Applications. 2003;13:343–379. doi: 10.1023/A:1022140919877

[44] Kaelbling LP. Hierarchical learning in stochastic domains: Preliminary results. In: Proceedings of the 10th International Conference on Machine Learning. San Mateo, CA: Morgan Kaufmann; 1993. p. 167–173.

[45] Bacon PL, Harb J, Precup D. The option-critic architecture. arXiv preprint arXiv:160905140. 2016;.

[46] Lima SL. Stress and decision making under the risk of predation: Recent developments from behavioral, reproductive, and ecological perspectives. Advances in the Study of Behavior. 1998;27:215–290. doi: 10.1016/S0065-3454(08)60366-6

[47] Brown JS. Vigilance, patch use and hahabit selection: Foraging under predation risk. Evolutionary Ecology Research. 1999;1:49–71.

[48] Brown JS, Kotler BP. Hazardous duty pay and the foraging cost of predation. Ecology Letters. 2004;7:999–1014. doi: 10.1111/j.1461-0248.2004.00661.x

[49] Blanchard RJ, Flannelly KJ, Blanchard DC. Defensive behaviors of laboratory and wild Rattus norvegicus. Journal of Comparative Psychology. 1986;100(2):101–107. doi: 10.1037/0735-7036.100.2.101 3720282

[50] Blanchard RJ, Blanchard DC. Antipredator defensive behaviors in a visible burrow system. Journal of Comparative Psychology. 1989;103(1):70–82. doi: 10.1037/0735-7036.103.1.70 2924531 10.1037/0735-7036.103.1.70

[51] Blanchard RJ, Blanchard DC, Rodgers J, Weiss SM. The characterization and modelling of antipredator defensive behavior. Neuroscience and Biobehavioral Reviews. 1990;14(4):463–472. doi: 10.1016/S0149-7634(05)80069-7 2287483 10.1016/s0149-7634(05)80069-7

[52] Bolles RC, Fanselow MS. A perceptual-defensive-recuperative model of fear and pain. Behavioral and Brain Sciences. 1980;3:291–323. doi: 10.1017/S0140525X0000491X

[53] Ydenberg RC, Dill LM. The economics of fleeing from predators. Advances in the Study of Behavior. 1986;16:229–249. doi: 10.1016/S0065-3454(08)60192-8

[54] Aupperle RL, Paulus MP. Neural systems underlying approach and avoidance in anxiety disorders. Dialogues in Clinical Neuroscience. 2010;12:517–531. 21319496 10.31887/DCNS.2010.12.4/raupperle PMC3181993

[55] McNaughton N, Corr PJ. A two-dimensional neuropsychology of defense: Fear/anxiety and defensive distance. Neuroscience & Biobehavioral Reviews. 2004;28:285–305. doi: 10.1016/j.neubiorev.2004.03.005 15225972 10.1016/j.neubiorev.2004.03.005

[56] Stephens DW. The logic of risk-sensitive foraging preferences. Animal Behaviour. 1981;29(2):628–629. doi: 10.1016/S0003-3472(81)80128-5

[57] Keramati M, Gutkin B. Homeostatic reinforcement learning for integrating reward collection and physiological stability. Elife. 2014;3:e04811 doi: 10.7554/eLife.04811 10.7554/eLife.04811 PMC4270100 25457346

[58] Charnov EL. Optimal foraging: The marginal value theorem. Theoretical Population Biology. 1976;9(2):129–136. doi: 10.1016/0040-5809(76)90040-X 1273796 10.1016/0040-5809(76)90040-x

[59] West M. Bayesian model monitoring. Journal of the Royal Statistical Society Series B (Methodological). 1986;48(1):70–78.

[60] Robbins TW. Cortical noradrenaline, attention and arousal. Psychological Medicine. 1984;14:13–21. doi: 10.1017/S0033291700003032 6709778 10.1017/s0033291700003032

[61] Nassar MR, Rumsey KM, Wilson RC, Parikh K, Heasly B, Gold JI. Rational regulation of learning dynamics by pupil-linked arousal systems. Nature Neuroscience. 2012;15(7):1040–1046. doi: 10.1038/nn.3130 22660479 10.1038/nn.3130 PMC3386464

[62] Preuschoff K, Marius’t Hart B, Einhäuser W. Pupil dilation signals surprise: Evidence for noradrenaline’s role in decision making. Frontiers in Neuroscience. 2011;5:115 doi: 10.3389/fnins.2011.00115 21994487 10.3389/fnins.2011.00115 PMC3183372

[63] Warren CM, Wilson RC, van der Wee NJ, Giltay EJ, van Noorden MS, Cohen JD, et al The effect of atomoxetine on random and directed exploration in humans. PLoS ONE. 2017;12(4):e0176034 doi: 10.1371/journal.pone.0176034 28445519 10.1371/journal.pone.0176034 PMC5405969

[64] Gilzenrat MS, Nieuwenhuis S, Jepma M, Cohen JD. Pupil diameter tracks changes in control state predicted by the adaptive gain theory of locus coeruleus function. Cognitive, Affective, & Behavioral Neuroscience. 2010;10(2):252–269. doi: 10.3758/CABN.10.2.252 10.3758/CABN.10.2.252 PMC3403821 20498349

[65] Trimmer PC, Houston AI, Marshall JA, Bogacz R, Paul ES, Mendl MT, et al Mammalian choices: Combining fast-but-inaccurate and slow-but-accurate decision-making systems. Proceedings of the Royal Society of London B: Biological Sciences. 2008;275:2353–2361. doi: 10.1098/rspb.2008.0417 10.1098/rspb.2008.0417 PMC2603220 18611852

[66] Koob GF. Corticotropin-releasing factor, norepinephrine, and stress. Biological Psychiatry. 1999;46(9):1167–1180. doi: 10.1016/S0006-3223(99)00164-X 10560023 10.1016/s0006-3223(99)00164-x

[67] McCall JG, Al-Hasani R, Siuda ER, Hong DY, Norris AJ, Ford CP, et al CRH engagement of the locus coeruleus noradrenergic system mediates stress-induced anxiety. Neuron. 2015;87(3):605–620. doi: 10.1016/j.neuron.2015.07.002 26212712 10.1016/j.neuron.2015.07.002 PMC4529361

[68] Hanes DP, Schall JD. Neural control of voluntary movement initiation. Science. 1996;274:427–430. doi: 10.1126/science.274.5286.427 8832893 10.1126/science.274.5286.427

[69] Frank MJ. Hold your horses: a dynamic computational role for the subthalamic nucleus in decision making. Neural Networks. 2006;19(8):1120–1136. doi: 10.1016/j.neunet.2006.03.006 16945502 10.1016/j.neunet.2006.03.006

[70] Frank MJ, Samanta J, Moustafa AA, Sherman SJ. Hold your horses: Impulsitivity, deep brain stimulation, and medication in Parkinsonism. Science. 2007;318:1309–1312. doi: 10.1126/science.1146157 17962524 10.1126/science.1146157

[71] Newell A, Simon HA. Human problem solving. Englewood Cliffs, NJ: Prentice-Hall; 1972.

[72] Russell SJ, Wefald EH. Do the right thing: Studies in limited rationality. MIT Press; 1991.

[73] Hansen EA, Zilberstein S. Monitoring and control of anytime algorithms: A dynamic programming approach. Artificial Intelligence. 2001;126(1-2):139–157. doi: 10.1016/S0004-3702(00)00068-0

[74] Dean T, Boddy M. An analysis of time-dependent planning. In: Proceedings of the Seventh National Conference on Artificial Intelligence. Morgan Kaufmann; 1988. p. 49–54.

[75] Zilberstein S. Operational rationality through compilation of anytime algorithms. AI Magazine. 1995;16(2):79–80.

